# Distal and Proximal Actions of Peptide Pheromone M-Factor Control Different Conjugation Steps in Fission Yeast

**DOI:** 10.1371/journal.pone.0069491

**Published:** 2013-07-16

**Authors:** Taisuke Seike, Taro Nakamura, Chikashi Shimoda

**Affiliations:** Department of Biology, Graduate School of Science, Osaka City University, Osaka, Japan; University of Cambridge, United Kingdom

## Abstract

Mating pheromone signaling is essential for conjugation between haploid cells of P-type (P-cells) and haploid cells of M-type (M-cells) in *Schizosaccharomyces pombe*. A peptide pheromone, M-factor, produced by M-cells is recognized by the receptor of P-cells. An M-factor-less mutant, in which the M-factor-encoding genes are deleted, is completely sterile. In liquid culture, sexual agglutination was not observed in the mutant, but it could be recovered by adding exogenous synthetic M-factor, which stimulated expression of the P-type-specific cell adhesion protein, Map4. Exogenous M-factor, however, failed to recover the cell fusion defect in the M-factor-less mutant. When M-factor-less cells were added to a mixture of wild-type P- and M-cells, marked cell aggregates were formed. Notably, M-factor-less mutant cells were also incorporated in these aggregates. In this mixed culture, P-cells conjugated preferentially with M-cells secreting M-factor, and rarely with M-factor-less M-cells. The kinetics of mating parameters in liquid culture revealed that polarized growth commenced from the contact region of opposite mating-type cells. Taken together, these findings indicate that M-factor at a low concentration induces adhesin expression, leading to initial cell-cell adhesion in a type of “distal pheromone action”, but M-factor that is secreted directly in the proximity of the adhered P-cells may be necessary for cell fusion in a type of “proximal pheromone action”.

## Introduction

Mating pheromones in ascomycete fungi play an important role in conjugation [1]. Yeast pheromones are simple peptides or modified peptides as found in *Saccharomyces cerevisiae* [2,3] and *Schizosaccharomyces pombe* [4,5]. One of the major functions of mating pheromones in *Saccharomyces* yeasts is to guide the mating projection to a cell of the opposite mating type [6]. A partner cell senses a gradient of pheromone and extends a mating projection towards the center of the pheromone source [7]. Another function of pheromones is thus to choose the most favorable partner who produces the pheromone in abundance [8]. Mating pheromones are specifically recognized by cognate receptors. Highly specific molecular recognition between a peptide pheromone and its cognate receptor serves as a barrier preventing interspecific hybridization, and thus plays an important role in reproductive isolation.

**Figure 1:**
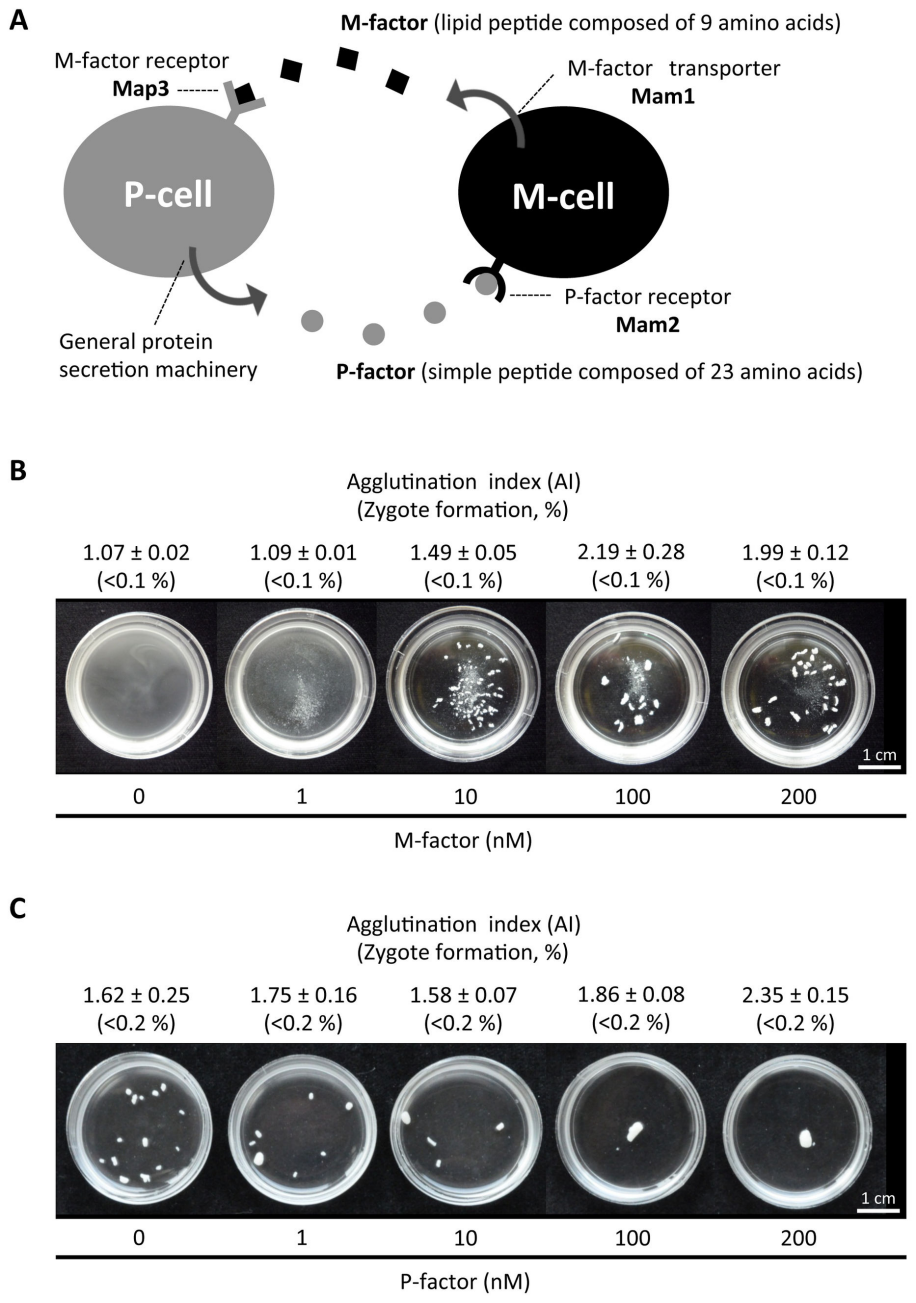
Induction of sexual agglutination by mating pheromone. (A) Illustration of mating pheromone signaling in *S. pombe*. The pheromone signal is transmitted through trimeric G-protein α-subunit and the mitogen-activated protein kinase cascade, comprising Byr2, Byr1 and Spk1. The signal transmission pathway is common to both M- and P-cells [22]. (B) The defect in sexual agglutination of the M-factor-less mutant was recovered by exogenously added synthetic M-factor. The homothallic strain FS55 was treated with synthetic M-factor for 4 hr in SSL−N. The agglutination index (AI) was determined for triplicate samples. Means with standard deviations are presented. A portion of each culture was incubated for 24 hr, and the frequency of zygotes was counted. At least 1,000 cells were examined for each sample. (C) Exogenous P-factor addition enhanced constitutive agglutination in a homothallic P-factor-less strain (FS251). At least 500 cells were observed for each sample.

In the fission yeast *S. pombe*, conjugation is induced between two different mating types, *h* ^+^ (P) and *h* ^–^ (M) [9–11]. Mating competence is attained by pheromonal communication between cells of the two different mating types. Pheromone signaling in *S. pombe* is illustrated in Figure 1A. P-cells secrete P-factor, a 23-amino-acid simple peptide, which is recognized by its cognate receptor, Mam2, on the cell surface of M-cells [12]. The mature P-factor peptide is processed from a precursor polypeptide encoded by the gene *map2* ^*+*^ [5]. M-cells produce M-factor, a nonapeptide whose C-terminal Cys residue is farnesylated and O-methylated [13,14]. M-factor is recognized by the Map3 receptor on P-cells [15]. Mature M-factor is encoded by triplicate redundant genes: *mfm1* ^*+*^, *mfm2* ^*+*^, and *mfm3* ^*+*^ [13,16]. Precursor proteins synthesized from these *mfm* genes are processed by as yet unidentified proteolytic enzymes to produce the same nonapeptide. Comprehensive mutagenesis has demonstrated that the primary sequence of the C-terminal half of M-factor is important for recognition by Map3 [17]. Both Mam2 and Map3 are heterotrimeric GTP-binding protein-coupled receptors containing 7 transmembrane domains. Activation of the associated Gα protein (Gpa1) transmits signals through the MAP kinase cascade, comprising Byr2/Ste8 (MAPKKK), Byr1/Ste1 (MAPKK) and Spk1 (MAPK), and finally induces transcription of a set of genes necessary for mating [18].

A pheromone-stimulated cell forms a projection from the cell tip that is directed toward a cell of the opposite mating type. Such polarized growth is controlled by a cell polarity regulator complex composed of Cdc42 (a Rab family GTPase), Scd1 (GEF for Cdc42) and Scd2 (a scaffold protein) [19]. On solid medium, cells of one mating type can sense a gradient of pheromone molecules secreted by cells of the opposite mating type, as suggested first in *Saccharomyces cerevisiae* [8,20]. Although similar mating projections are formed in liquid medium, the mechanism by which the mating partner is sensed remains to be elucidated.

In nature, fission yeasts are thought to live in a semi-aqueous environment. Because a pheromone gradient is unlikely to be formed in liquid culture, polarized growth of the projection might be controlled by a totally different mechanism. Prior to cell fusion, cell adherence occurs between opposite mating-type cells, resulting in macroscopic cell agglutination [21], which may help the cells to find their mating partners. Sexual cell adhesion is attained by two mating-type-specific adhesin glycoproteins, Map4 and Mam3 [22,23]. Because cell fusion rarely occurs in adhesin-deficient mutants, cell-to-cell contact within the cell aggregates must be necessary for cell fusion between mating partners.

M- and P-cells are mutually stimulated by mating pheromones. Notably, M-factor production is induced solely by nitrogen-starvation and does not need stimulation by P-factor, whereas P-factor expression is enhanced by M-factor [22]. These observations imply that M-factor signaling takes the initiative in the pheromonal control of mating. In this study, we have focused our attention on M-factor signaling and on two different modes of action of M-factor—namely its distal and proximal actions—in the mating process. We have also attempted to trace the polarized growth in liquid culture that leads to cell fusion, a step that is controlled by the proximal (or direct) mode of action of M-factor on P-cells, focusing on the role of cell-to-cell contact, a step that is triggered by the distal action of M-factor in culture fluid.

## Materials and Methods

### Yeast strains, media and culture conditions

The *S. pombe* strains used in this study are listed in Table 1. Standard methods were used for growth, transformation, and genetic manipulation [24]. *S. pombe* cells were grown on yeast-extract agar medium (YEA) supplemented with adenine sulfate (75 mg/l), uracil (50 mg/l) and leucine (50 mg/l). Antibiotics (G418, Hygromycin B and Nourseothricin) were added to YEA medium at a final concentration of 100 µg/ml. Minimal medium SD was used for selection of auxotrophic mutants. Other synthetic media, MM+N and MM−N, were used for overexpression of manipulated genes. Solid media used for mating and sporulation were MEA and SSA. For induction of mating in liquid media, a shift from SSL+N to nitrogen-free medium (SSL−N) was applied [9,25]. Cells were incubated at 30^o^C for growth and at 28^o^C for mating, unless stated otherwise.

**Table 1:** Strains used in this study.

**Strains**	**Genotype**	**Source**
L968	*h* ^*90*^	Leupold U
L972	*h* ^*–*^	Leupold U
L975	*h* ^*+*^	Leupold U
Eg928	*h* ^*90*^ * mam1::ura4* ^*+*^ * ura4-D18*	Nielsen O
FS55^a^	*h* ^*90*^ * mfm1::LEU2 mfm2-D4 mfm3::ura4* ^*+*^ * ade6-M210 ura4-D18 leu1-32*	[17]
FS65	*h* ^*–*^ * mam2::LEU2 leu1*	This study
FS66	*h* ^*+*^ *map3* *::ura4* ^*+*^ * ura4-D18*	This study
FS71	*h90 * *map4* *::ura4* ^*+*^ * ade6-M210 ura4-D18 leu1*	This study
FS85	*h* ^*+*^ * ade6-M210 ura4-D18 leu1*	This study
FS103	*h* ^*+*^ * map4::ura4* ^*+*^ * map4:leu1* ^*+*^ *:map4-GFP ade6-M210*	This study
FS114	*h* ^*+*^ * ste11::ura4* ^*+*^ * ade6-M210 ura4-D18 leu1*	This study
FS120	*h* ^*–*^ * mfm1::LEU2 mfm2-D4 mfm3::ura4* ^*+*^ * ade6-M210*	This study
FS121	*h* ^*–*^ * ade6-M210*	This study
FS123	*h* ^*90*^ * fus1::kanR ade6-M216 leu1*	This study
FS127	*h* ^*+*^ * ura4-D18*	This study
FS133	*h* ^+^ *ade6-M210 ura4-D18 leu1* [pTA14]	This study
FS135	*h* ^+^ *ade6-M210 ura4-D18 leu1* [pTA14(map4^PRO^-lacZ)]	This study
FS251	*h* ^*90*^ * map 2::ura4* ^*+*^ * ade6-M216 ura4-D18 leu1-32*	This study
FS357	*h* ^*–*^ * ChrI:o14-kanR*	This study
FS403	*h* ^+^ *ste11::ura4* ^*+*^ ade6-M210 ura4-D18 leu1 [pTA14(map4^PRO^-lacZ)]	This study
FS404	*h* ^*+S*^ * ade6-M210 ChrII:d16-natR*	This study
FS419	*h* ^*–*^ * mfm1::LEU2 mfm2-D4 mfm3::ura4* ^*+*^ * ade6-M216 ura4-D18 leu1-32 ChrI:map3* ^*+*^ *-hygR*	This study
FS423	*h* ^*–*^ * ChrI:map3* ^*+*^ *-hygR*	This study
SS1290	*h* ^90^ *mfm1::LEU2 mfm2-D4 mfm3::ura4* ^+^ *ade6-M210 ura4-D18 leu1-32* [pSLF173(map4)]	This study
SS1463	*h* ^*–*^ * mam2::LEU2 mam3-mCherry-kanR leu1*	This study

The *S. pombe* strains constructed in this study will be deposited in the Yeast Genetic Resource Center of Japan, which is supported by the National BioResource Project YGRC/NBRP. ^a^ This strain was obtained from YGRC/NBRP (http:/yeast.lab.nig.ac.jp/nig/).

### Quantitative assay of zygote formation and sexual cell agglutination

Cells grown on YEA were inoculated into SSL+N liquid medium at a cell density of 2×10^6^ cells/ml. Cultures were shaken at 30^o^C overnight. A 3–5 ml batch of SSL−N was then inoculated with the cells at a density of 4×10^7^ cells/ml, and cultured at 28^o^C with shaking (at 200 rpm).

The frequency of zygotes including asci was determined by light microscopy. Usually, triplicate samples (at least 250 cells each) were counted. Cell types were classified into four groups: vegetative cells (V), zygotes (Z), asci (A) and free spores (S). The percentage of zygotes was calculated according to the following equation: *Zygotes* (*%*) *=* (*2Z+2A+S/2*) *×* 100/*(V*+2*Z*+2A+*S*/2*)*

The intensity of sexual cell agglutination was photometrically estimated according to Shimoda and Yanagishima [26]. Sexual cell agglutination was induced by shifting from SSL+N to SSL−N, as mentioned above. An aliquot of the culture was diluted with water, and the optical density at 600 nm was measured (OD_600_ before sonication). The cell suspension was then subjected to sonication (20 kHz) for 10 sec to disperse cell aggregates completely. Immediately after sonication, the OD_600_ was determined (OD_600_ after sonication). The agglutination index (AI) was calculated according to the following equation: *AI =OD600* (*after sonication*) */ OD*_*600*_ (*before sonication*)

This value has been shown to be a function of mean size of cell aggregates [27].

### Quantitative assay of hybrid formation by recombinant frequency

Mating efficiency was also deterimined by a quantitative assay of genetic recombination. Heterothallic haploid strains carrying drug-resistance markers (*natR*, *kanR* and *hygR*) on the chromosomes (Seike et al., in preparation) were mixed and cultured in SSL−N medium for 2 days. The cell suspension was diluted and spread on different drug-containing plates. The number of colonies was counted after 3 days of incubation.

### Synthetic M-factor and P-factor peptides

M-factor peptides were chemically synthesized basically according to Wang et al. [28] (Peptide Institute Inc., Osaka, Japan). The purity of the preparation was over 96%. P-factor peptides were also chemically synthesized (Operon, Tokyo, Japan). M-factor and P-factor were dissolved in methanol at a concentration of 2 mg/ml. These stock solutions were diluted with culture medium in the appropriate dilution ratio.

### Construction of a *map4* promoter-*lacZ* fusion gene

A pDB248’-based multicopy plasmid carrying the *mat 1-Pm/lacZ* fusion gene named pTA14 [29] was reconstructed to carry a *map4* promoter-*lacZ* fusion construct. In brief, a *Xho*I/*Hin*dIII fragment containing the mat 1-Pm promoter sequence was replaced by a 603-bp *Xho*I/*Hin*dIII fragment containing the putative *map4* promoter sequence. The resulting plasmid, pTA(map4^PRO^-lacZ), was transformed into heterothallic *h* ^+^ strains, FS114 and FS133. Additionally, a few deletion sets of the *map4* promoter were constructed by PCR. The primer sequences are presented in Table S1.

Expression of map4^PRO^-lacZ was assayed in two transformants (FS135 and FS403) by β-galactosidase activity. To avoid loss of the pTA(map4^PRO^-lacZ) plasmid, transformants were maintained on SD medium lacking leucine. After permeabilization [30], β-galactosidase activity was assayed using ONPGal (o-nitrophenyl-β-D- galactopyranoside) as a substrate [31].

### Expression of Map4-GFP and Mam3-mCherry

Expression of Map4 was examined by fluorescence intensity of a *map4*-GFP (green fluorescent protein) fusion gene, which was chromosomally integrated at the authentic *map4* locus in the *map4::ura4* ^+^ disruption background [23]. Similarly, a *mam3*-mCherry fusion gene was integrated at the authentic locus. Fluorescence intensity of cells was analyzed by Image-J software (http://rsbweb.nih.gov/ij/features.html).

### Observation of cell morphology

Fluorescent signals of GFP and mCherry were observed under a fluorescence microscope (BX-51, Olympus Co., Tokyo, Japan). Digital images obtained by a differential interference contrast microscope were recorded. Microphotographs were analyzed for the frequency of zygotes and prezygotes (paired cells in contact at projection tips). The long axis (L) and the short axis (S) of a cell were measured, and the L/S ratio was calculated as a measure of cell shape.

## Results

### The primary action of M-factor is induction of sexual agglutination

Conjugation of *S. pombe* in liquid medium starts with sexual cell agglutination in response to mating pheromone secreted by cells of the opposite mating type [21]. To investigate the biological roles of M-factor pheromone in the conjugation process, we used an M-factor-less (*mfm1,2,3Δ*) mutant, FS55 (Table 1) [16], in a cell agglutination assay. This homothallic strain showed no cell agglutination, suggesting that M-factor is essential for cell-cell adhesion between P- and M-cells. The M-factor-less mutant was treated with synthetic M-factor at different concentrations (0–200 nM) in nitrogen-free medium (SSL−N). Exogenously added M-factor clearly induced cell agglutination at concentrations higher than 10 nM after 4 hr of incubation (Figure 1B). This agglutination assay is an extremely sensitive test for the biological activity of M-factor; thus, we conclude that M-factor secreted by M-cells renders P-cells adhesive to M-cells. As a control, a wild-type homothallic strain (L968) was treated with various concentrations of M-factor (Supplementary Figure S1A). Strong agglutination occurred without exogenous M-factor, and the addition of M-factor did not markedly enhance the intensity of agglutination. The intensity of sexual cell agglutination in wild-type cells that were not treated with exogenous M-factor was comparable to that in M-factor-less cells treated with exogenous M-factor at 10 nM.

### Constitutive expression of M-cell adhesibility

Next, we tested whether the sexual agglutination of M-cells required P-factor pheromone. A homothallic strain (FS251) harboring a deletion of the *map2* ^*+*^ gene encoding P-factor pheromone was cultured in nitrogen-free medium containing various concentrations of synthetic P-factor (0–200 nM) for 4 hr. The P-factor-less cell population exhibited significant agglutination without exogenous P-factor, and the addition of synthetic P-factor further enhanced the intensity of agglutination (Figure 1C). We thus conclude that stimulation of cell adhesibility by P-factor is auxiliary, and that M-factor-induced agglutination of P-cells is critical for the commencement of cell-to-cell contact. Indeed, the transcription of *mam3* ^*+*^, encoding an M-type-specific adhesin protein, is known to be induced substantially under nitrogen starvation without pheromone signaling, whereas the mRNA expression of *map4* ^*+*^, encoding a P-type-specific adhesin protein, requires pheromone signaling [22].

**Figure 2:**
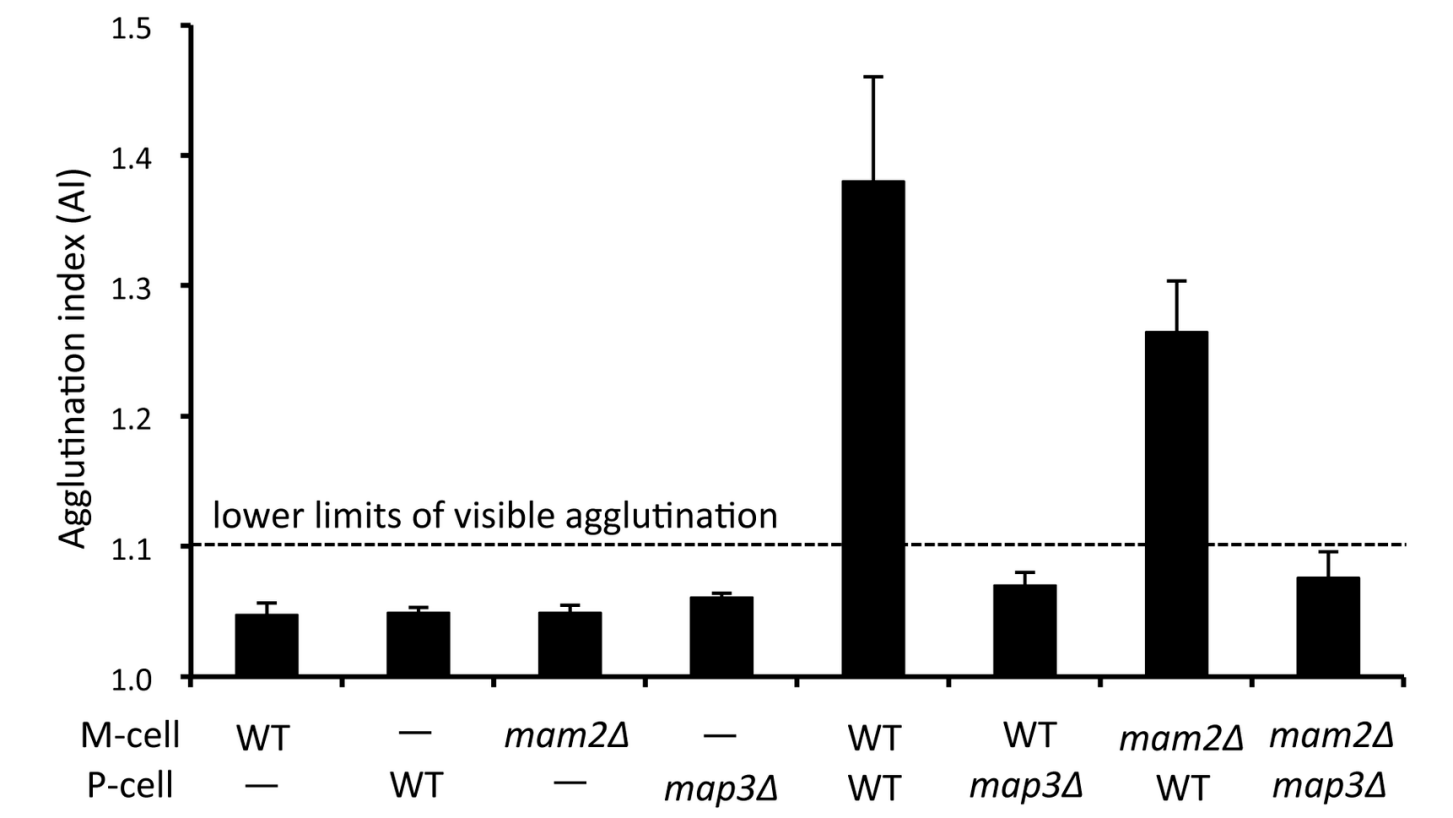
Agglutination ability of heterothallic strains harboring deletion of the pheromone receptor gene. The strains used were L972 (*h* ^–^ wild type), L975 (*h* ^+^ wild type), FS65 (*h* ^*–*^ *mam2Δ*), and FS66 (*h* ^*+*^ *map3Δ*). Cells were incubated with gentle shaking in SSL−N for 4 hr. AI was determined for triplicate samples. Means with standard deviations (vertical bars) are presented. Visible agglutination was seen in samples with an AI of more than 1.1.

**Figure 3:**
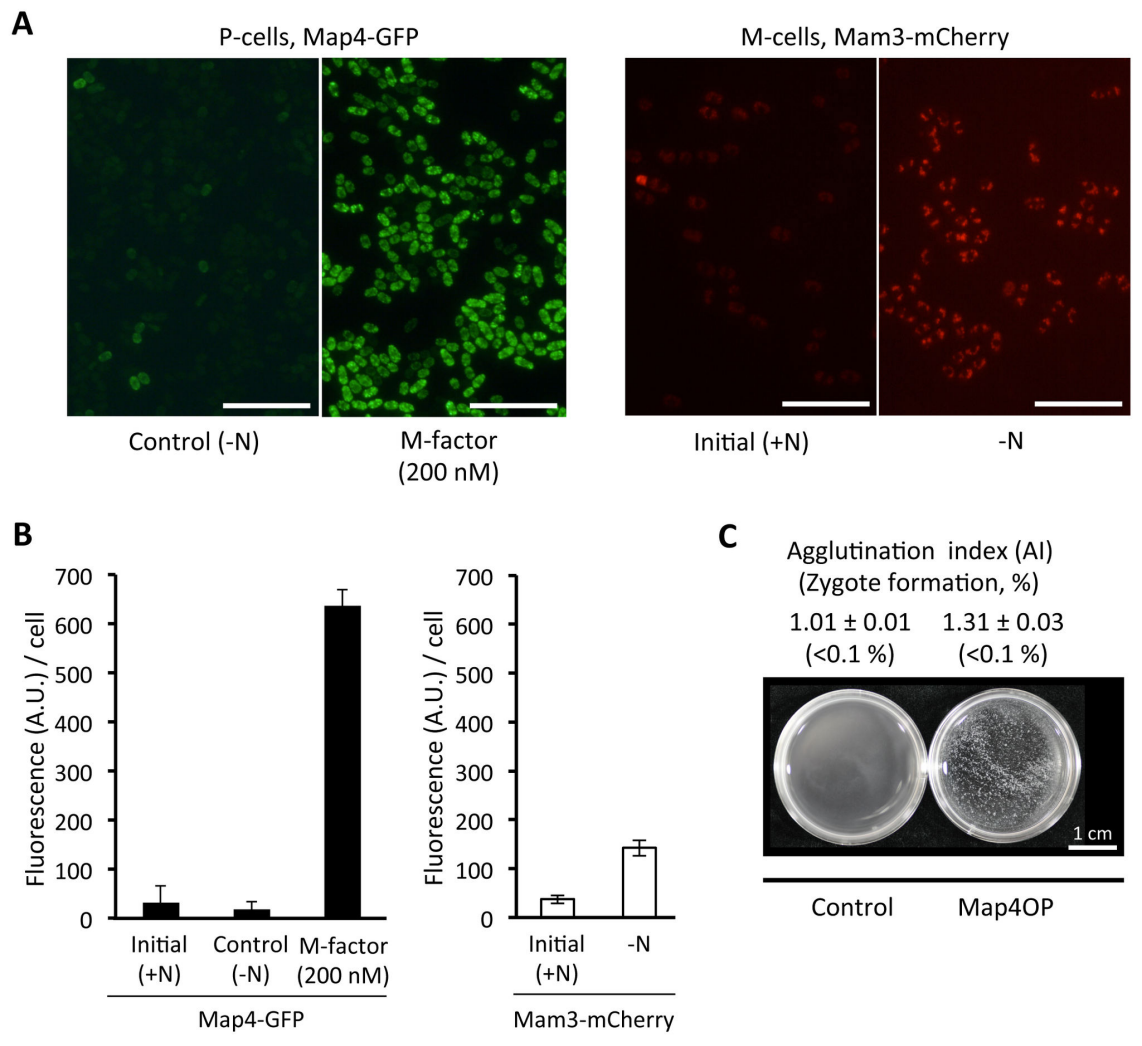
Expression of mating-type-specific adhesin proteins, Map4 and Mam3 (A, left). A heterothallic *h* ^+^ strain (FS103) carrying a *map4*-*GFP* fusion gene integrated at the authentic chromosomal locus was precultured in SSL+N for 18 hr, then shifted to SSL−N with or without 200 nM of M-factor, and cultured for 4 hr. (A, right) A heterothallic *h* ^−^ *mam2Δ* strain (SS1463) carrying a *mam3-mCherry* fusion gene integrated at the original chromosomal locus was incubated for 4 hr in SSL−N (−N). Scale bar, 50 µm. (B) Quantification of Map4-GFP and Mam3-mCherry fluorescence. The intensity was quantified by image analysis using Image-J software. At least 8 different areas were analyzed. Means of arbitrary units (A.U.) with standard deviations are presented. (C) Induced agglutination by overexpression of Map4. Strain SS1290 ectopically overexpressing *map4* ^+^ driven by the *nmt1* promoter was precultured in MM+N without thiamine for 12 hr and then cultured in MM−N for 4 hr (Map4OP). The same strain was transformed by the pSLF173 empty vector [43] as a control.

Furthermore, we assayed the cell agglutination of heterothallic M- and P-type strains carrying a disrupted gene for *map3* or *mam2*, encoding the M-factor or P-factor receptor, respectively (Figure 2). As expected, *map3Δ* P-cells were unable to agglutinate with wild-type M-cells. Interestingly, however, *mam2Δ* M-cells exhibited significant agglutination with wild-type P-cells, suggesting that P-factor signaling is not essential for the induced cell adhesibility of M-cells. These observations are consistent with transcription of the *mam3* ^*+*^ gene, which is partially induced by nitrogen starvation and further enhanced by P-factor signaling [22]. On the other hand, *map4* transcription is fully dependent on the presence of M-factor in nirogen-free medium [22]. To confirm the results of the previous transcription analysis, we quantified the expression level of Mam3 protein. Expression of Mam3-mCherry fluorescence was examined in a heterothallic *h* ^–^ *mam2Δ* strain. As shown in Figure 3A, significant fluorescence from Mam3-mCherry was detected when the cells were shifted cells to nitrogen-free medium. Treatment with P-factor did not enhance the fluorescence level (data not shown). It is possible that P-factor might be degraded by Sxa2 protease [32]. In conclusion, M-cells become adhesive in pheromone-free nitrogen-starved medium, but P-cells have no adhesibility without M-factor. As reported previously [12,15], both the *mam2Δ* and *map3Δ* mutants are sterile. Taken together, these results show that P-factor signaling is not essential for cell agglutination, but is indispensable for cell fusion leading to zygote formation.

### The P-cell-specific adhesin Map4 is upregulated in response to M-factor

Mating-type-specific cell adhesion is mediated through molecular interactions between cell-surface adhesin glycoproteins of M-type (Mam3) and P-type (Map4) cells [22,23]. It has been shown that the *map4* ^+^ mRNA level is dependent on mating pheromone signaling [22] and that Map4 protein expression is limited to P-cells. We therefore predicted that the induction of sexual cell adhesion that we observed in Figure 1 was critically triggered by the production of Map4. To verify that the production of Map4 protein is induced in response to M-factor treatment, a heterothallic P-type strain (FS103), harboring a *map4*-GFP allele at the authentic chromosomal locus [23], was used. This strain was treated with 200 nM synthetic M-factor, which induced cell agglutination, in SSL−N liquid medium. Aliquots were sampled at 4 hr, the cells were examined under a fluorescence microscope (Figure 3A), and the fluorescence intensity due to Map4-GFP was determined by Image-J software. As shown in Figure 3B, M-factor treatment greatly enhanced the fluorescence intensity, and thus the expression of Map4-GFP in 4 hr. We conclude that M-factor induced the production of Map4 in P-cells. We next assessed whether Map4 expression is sufficient for sexual agglutination induced by M-factor. Map4 was overexpressed via an nmt1 promoter in the M-factor-less strain SS1290. Ectopic overproduction of Map4 caused moderate agglutination, but no zygote formation was detected (Figure 3C), indicating that induction of Map4 is the central factor in pheromone-induced cell agglutination. Conversely, treatment of *map4Δ* cells with exogenous M-factor at 1–200 nM failed to cause sexual agglutination (Figure S1C). These experiments indicate that Map4 is a major molecular target of M-factor action on sexual cell agglutination.

**Figure 4:**
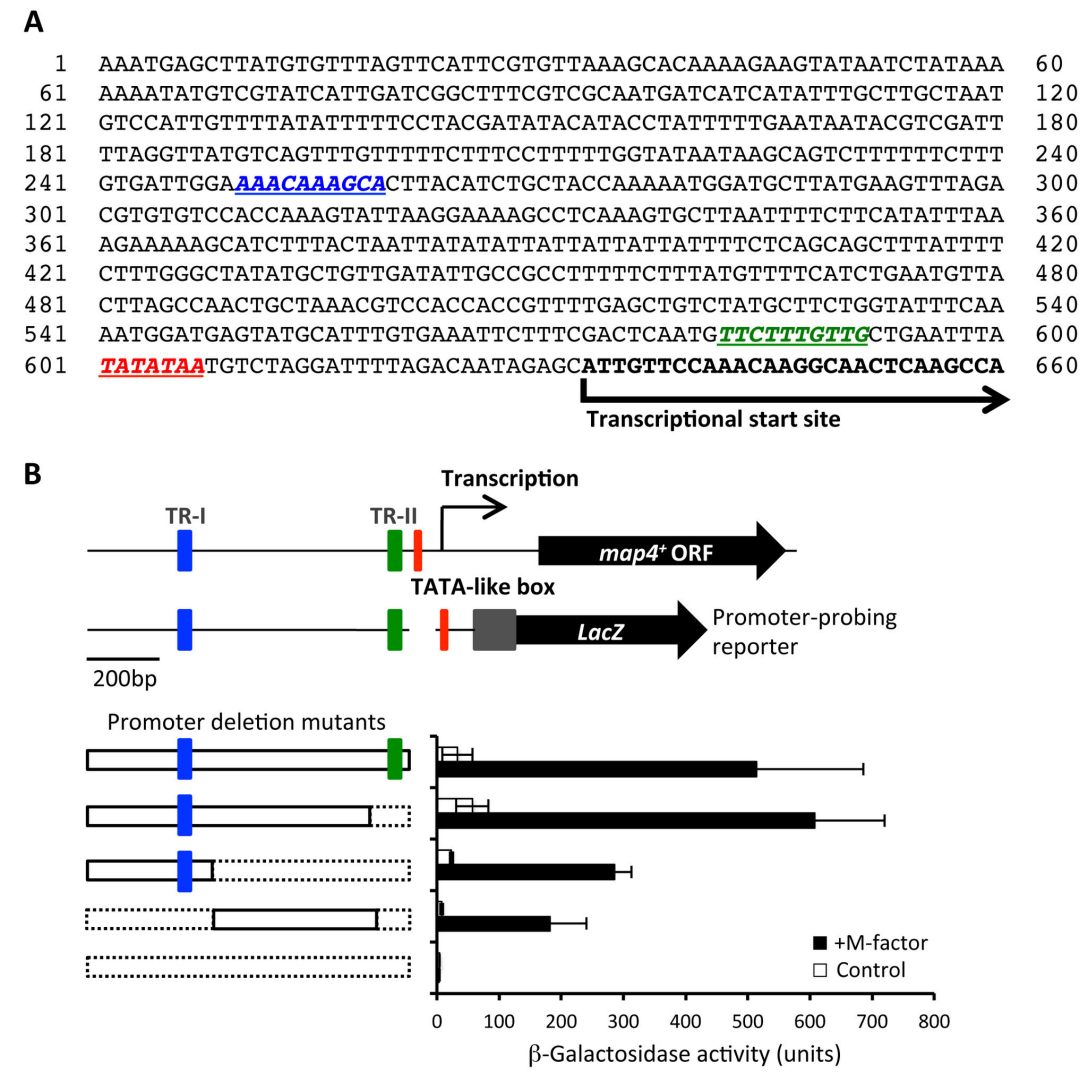
Deletion analysis of upstream *cis*-acting elements of *map4* ^+^. (A) Putative promoter sequence of the *map4* ^*+*^ gene. Two possible TR-box elements (blue and green) and a TATA-like box (red) are italicized and underlined. The transcription start site was identified by sequencing of several cDNA clones (Nakamura *et al*, unpublished). (B) β-galactosidase assay of the promoter intensity of a *map4* promoter-*lacZ* fusion gene with different deletions of the promoter sequence of the *map4* gene. A heterothallic *h* ^+^ strain (FS85) was transformed with the multicopy plasmid pTA(map4^PRO^-lacZ) or the deletion constructs. Cells of the transformants were precultured in SSL+N without leucine and then transferred to SSL−N medium containing 200 nM of synthetic M-factor (filled bar) or SSL−N alone (open bar). After 6 hr, β-gal activity was assayed.

Transcription of *map4* ^*+*^ has been reported to be dependent on Ste11, which is a key transcription factor for sexual reproduction [33]. A canonical 10-mer sequence (TTCTTTGTTY), called the TR-box motif, is the binding site for Ste11 [34], and we noted that there are two TR box-like motifs in the putative promoter sequence of *map4* ^*+*^ (Figure 4A). To verify the importance of these putative Ste11-binding sites in the promoter region of *map4* ^*+*^, several deletion constructs of this region were created (Figure 4B). First, we generated a map4^PRO^-lacZ fusion construct, and verified that β-galactosidase (β-gal) activity from this construct was not detectable in a *ste11Δ* mutant (Supplementary Figure S2). The plasmid pTA(map4^PRO^-lacZ) carrying various deletions of the *map4* promoter region was then introduced into a heterothallic P-type strain, FS103. For each construct, three independently isolated transformants were cultured in SSL−N medium with or without 200 nM M-factor. After 6 hr of incubation, β-gal activities were assayed (Figure 4B). The *lacZ* gene with a full-length *map4* promoter (ca. 600-bp) gave a high level of β-gal activity in the presence of M-factor. Complete deletion of the *map4* promoter sequence resulted in null activity. Our previous promoter analysis of another M-factor responsive gene, *mat 1-Pm* ^*+*^, showed that a TR-box immediately upstream of the TATA-box is critical for expression [29]. In the *map4* promoter, the most proximal TR-box (TR-II) is positioned 10 nucleotides upstream of the putative TATA box. Deletion of the proximal 100-bp stretch encompassing TR-II did not cause a reduction in β-gal activity. Additional deletion of a distal 250-bp sequence including a TR-box (TR-I) reduced the β-gal activity to about 40% of the full-length positive control. Thus, deletion of the putative TR-box elements did not result in a complete loss of *map4* expression. These deletion analyses indicated that the two TR box elements are not critical for the expression of Map4.

### M-factor-less cells are also incorporated in cell aggregates

**Figure 5:**
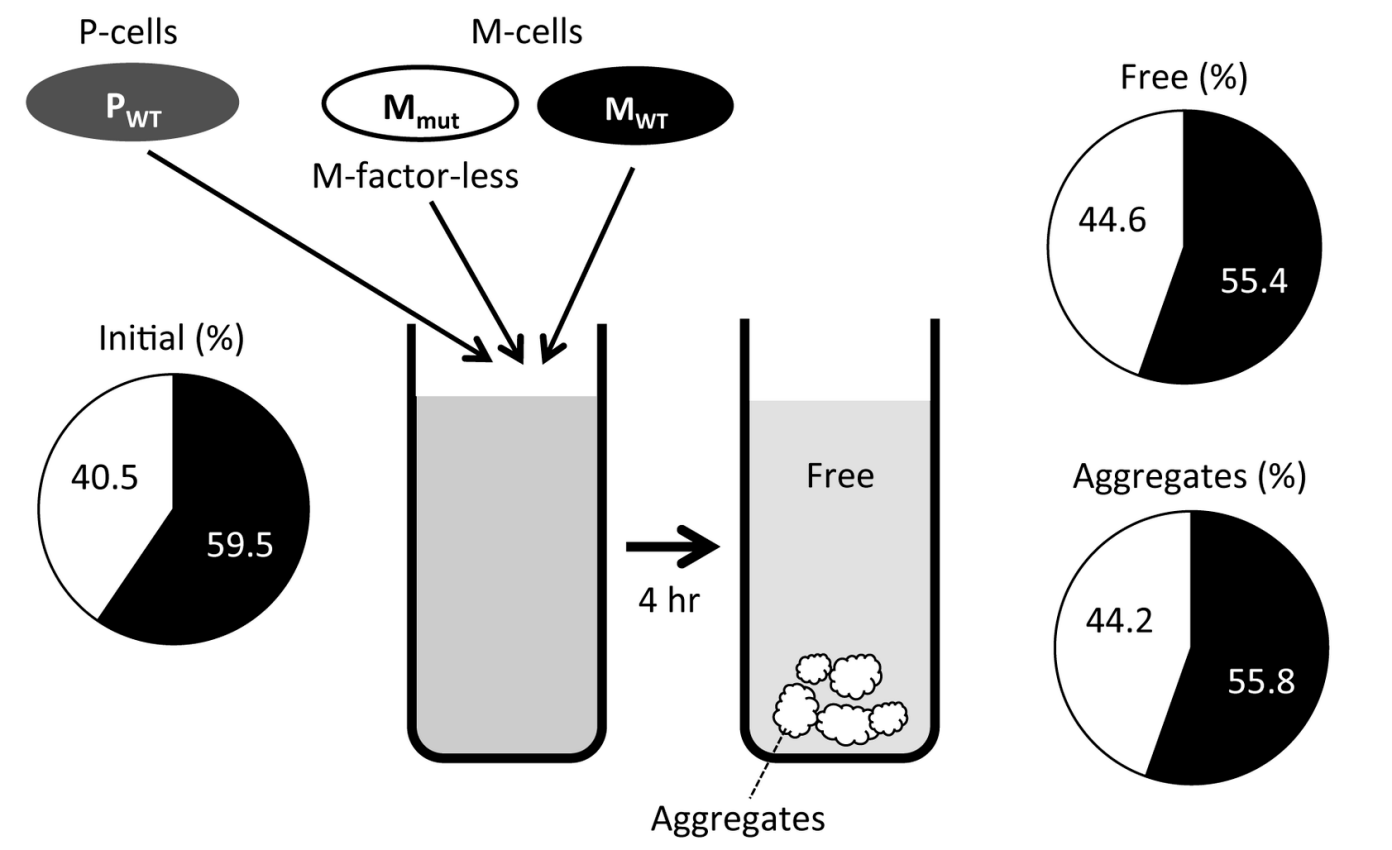
M-factor-less cells are incorporated in cell aggregates during co-cultivation with wild-type M-cells. Wild-type P-cells (FS127), wild-type M-cells (L972) and M-factor-less M-cells (FS120) were mixed in SSL−N in the cell number ratio of 2:1:1. FS127 carries a uracil-auxotrophic marker (*ura4-D18*), and FS120 harbors an adenine-auxotrophic marker (*ade6-M210*). After 4 hr of culture with shaking, cell aggregates were obtained after the culture was allowed to stand for a few minutes. Cells were sampled from the precipitated aggregates and the floating fractions separately. The fractions were briefly sonicated to disperse cell clusters, and the cells were spread on SD without leucine but with one-quarter strength adenine-sulfate. P-cells could not form colonies because of uracil depletion. M-cells derived from the M-factor-less strain formed red colonies due to *ade6-M210*, and those from the wild-type strain formed white colonies.

As described above, homothallic M-factor-less mutant (FS55) cells are unable to agglutinate, but this defect is phenotypically suppressed by exogenous M-factor (Figure 1B). We therefore predicted that M-factor-less M-cells might adhere to wild-type P-cells if an M-factor producer (i.e. wild-type M-cells) were added to the same culture flask. We performed a mixed culture of three cell types, M-factor-less M-cells, wild-type M-cells and wild-type P-cells, at an initial cell number ratio of 1:1:2. In this culture, strong cell agglutination was observed (data not shown). After 4 hr of incubation, the culture was allowed to stand for a few minutes to enable large cell aggregates to settle to the bottom. Floating free cells were then separated from the pellet with a pipette. Cells of the two fractions, a floating fraction and a pelleted aggregate, were briefly sonicated and spread on uracil-free minimal medium to avoid colony formation by P-cells. M-factor-less cells were marked by *ade6-M210* allele, which leads to red-colored colonies on adenine-limited medium. We counted the number of white colonies (M-cells derived from the wild-type strain) and red colonies (M-cells derived from M-factor-less strain) on solid medium. As shown in Figure 5, the ratio of wild-type M-cells and M-factor-less M-cells was roughly the same (ca. 55:45) in both the aggregating and free cell fractions. This observation revealed that even M-factor-less cells are efficiently incorporated into the cell aggregates.

### Pheromone-less M-cells are unable to mate with P-cells even in presence of exogenous M-factor

**Figure 6:**
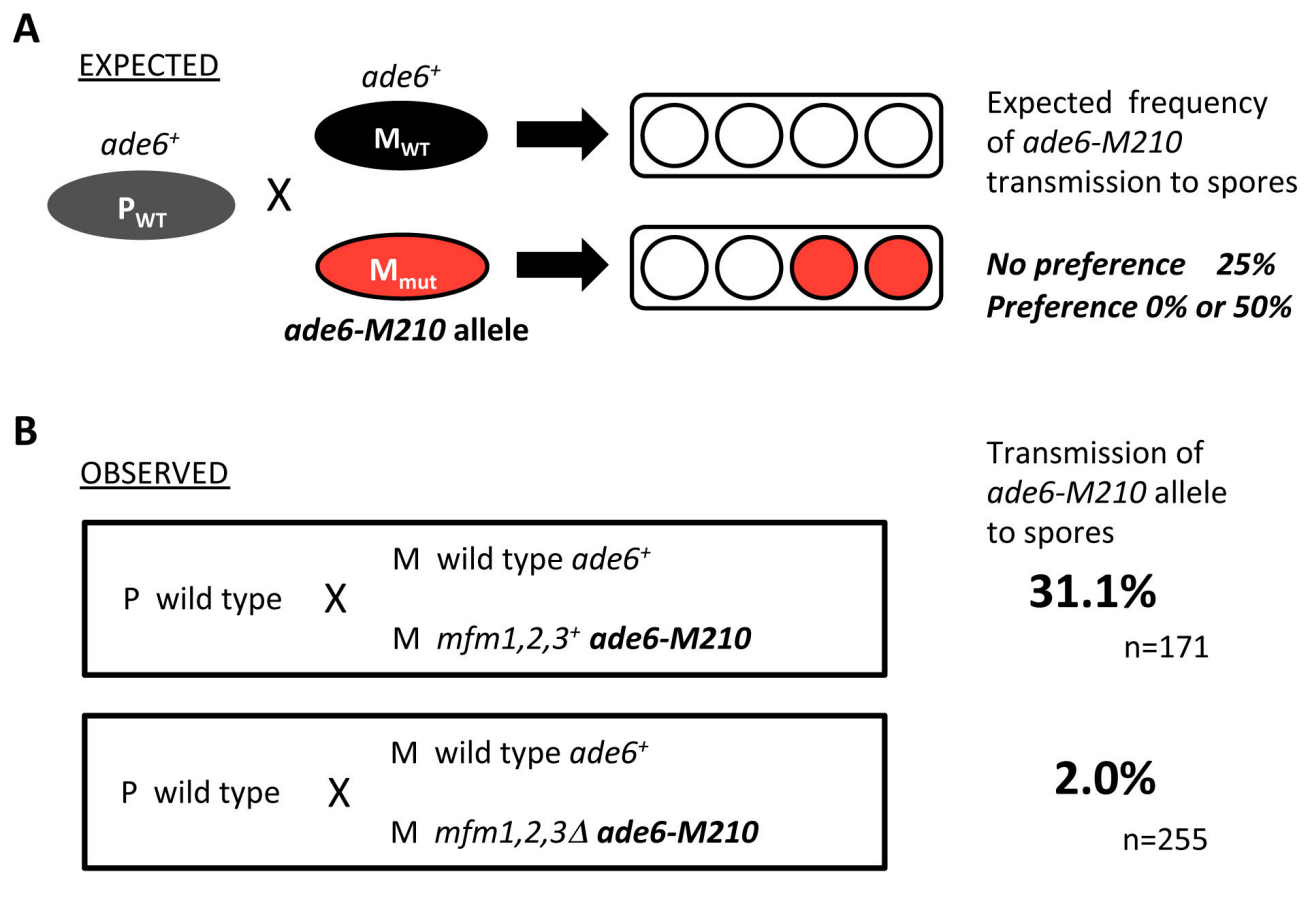
P-cells preferentially mate with wild-type M-cells. (A) Experimental design. Wild-type P-cells (P_WT_) were mixed with an equal number of wild-type M-cells (M_WT_) and M-factor-less M-cells (M_mut_), marked by the *ade6-M210* auxotrophic marker, in nitrogen-free medium. If the P-cell chose an *ade6* ^*+*^ M-cell, the descendant spore clones would have no *ade6-M210* allele. If the P-cell chose an *ade6-M210* M-cell, the allele would be transmitted to half of the descendant spore clones. Thus, the preference of P-cells for type of mating partner could be determined by the frequency of *ade6-M210* segregants. (B) Experimental results. The following strains were used: L972 (*h* ^–^ prototrophic), L975 (*h* ^+^ prototrophic), FS120 (*h* ^*–*^ *mfm1,2,3Δ ade6-M210*), and FS121 (*h* ^–^ *mfm1,2,3* ^+^ *ade6-M210*). The mixed cells were allowed to mate, and resulting hybrid diploids were sporulated. Spores were isolated by micromanipulation and grown on nutrient medium with adenine sulfate limitation.

As described above, the defect in cell adhesion of M-factor-less M-cells was recovered by adding exogenous synthetic M-factor; however, exogenous M-factor did not rescue the cell fusion defect (Figure 1B), as Nielsen’s group has already observed [16]. Similarly, the sterility of *map2Δ*, harboring a deletion of *map2* ^+^ encoding the counterpart pheromone P-factor [5], was not suppressed by the addition of synthetic P-factor (Figure 1C). Normal conjugation is attained by the down-regulation mechanism of P-factor, in which the specific protease, Sxa2, is involved [32]. Although the counterpart of Sxa2 for M-factor has not been identified, the control of M-factor concentration must also be important. We considered that the cell fusion defects may be due to fine tuning of the concentration of pheromones and the timing of pheromone addition. To verify this possibility, we conducted mixed culture experiments in which wild-type and M-factor-less M-cells, as well as wild-type P-cells, were inoculated into nitrogen-free medium (SSL−N) at a cell number ratio of 1:1:2. The M-factor-less M-type strain was genetically marked with the *ade6-M210* allele. The mixed cells were cultured overnight to support mating. A pair of M- and P-cells conjugate to form a zygote, which then culminates in an ascus containing four spores. In the mixed culture, two different kinds of zygotes might be formed: one hybrid between a wild-type P-cell and a wild-type M-cell, and the other hybrid between a wild-type P-cell and an M-factor-less M-cell. In the latter type of zygote, the ascus should produce two spores harboring the *ade6-M210* allele. In the case that P-cells mate equally with wild-type and mutant M-cells, the frequency of spores carrying the *ade6* allele is predicted to be 25% (i.e. two of the eight spores) (Figure 6A). In the case that P-cells prefer wild-type M-cells, the frequency of mutant M-spores is expected to be less than 25% (Figure 6A).

Spores were isolated by micromanipulation and incubated on YE nutrient medium without supplemental adenine sulfate. Spore-derived colonies remained white (*ade6* ^+^) or turned red (*ade6* ^*-*^), and the white and red colonies were counted (Figure 6B). Transmission of the *ade6-M210* allele to spores was less than 2%, which is significantly lower than 25%, indicating the preference of P-cells for wild-type M-cells. As a control, two wild-type strains of M-cells, one of them carrying *ade6-M210*, were mixed with a P-type strain. In this case, transmission of *ade6-M210* was 31%, which is close to the predicted value of 25%.

**Figure 7:**
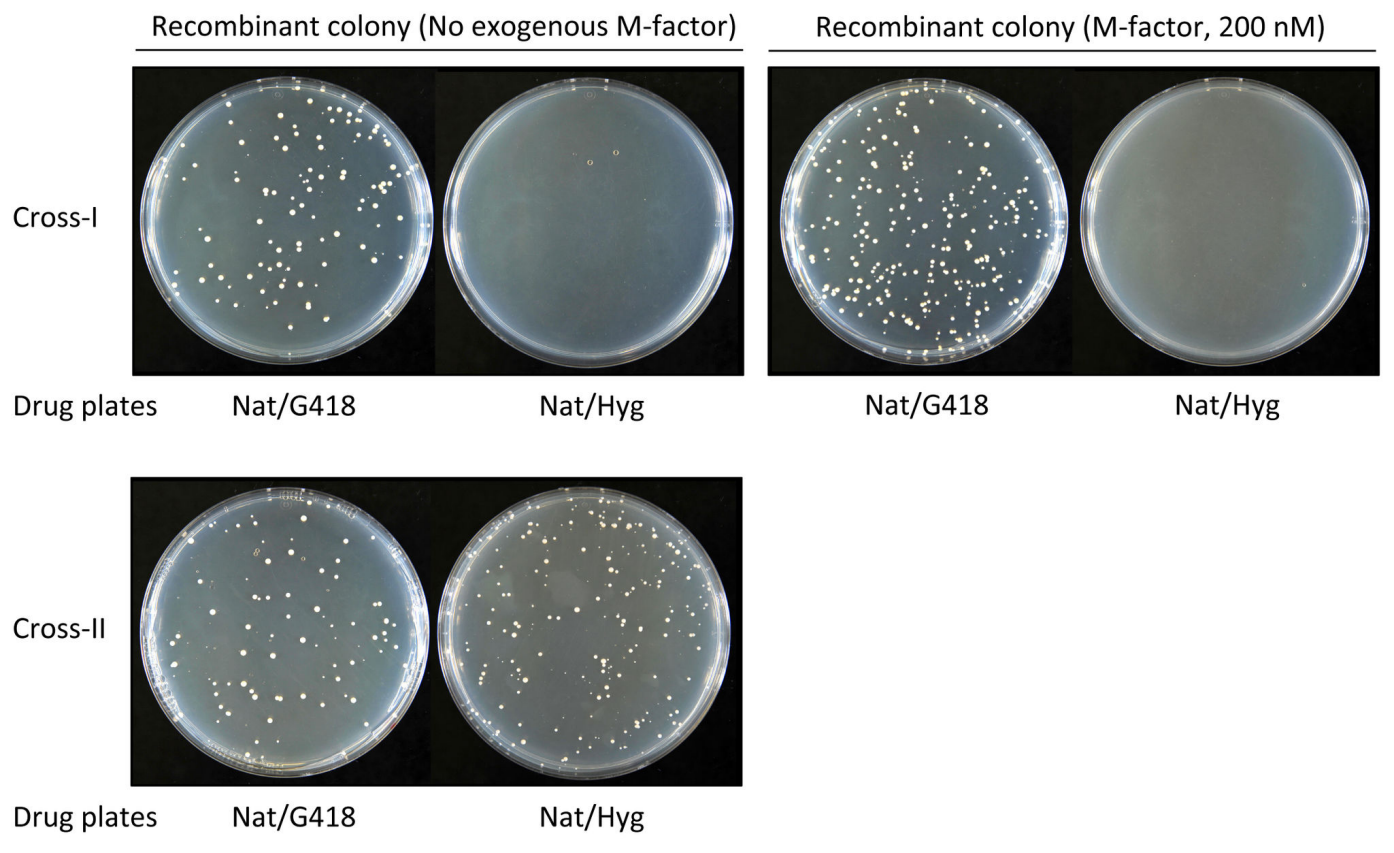
Recombinant frequency assay of wild-type and pheromone-less mutant strains. Heterothallic haploid strains carrying a *natR*, *kanR* or *hygR* drug-resistant marker were cultured in SSL+N overnight and then mixed in the nitrogen-free conjugation medium (SSL−N) with or without M-factor (200 nM), and cultured at 28^o^C with shaking. Two different mixed cultures ran in parallel. Cross-I, P-type strain (FS404; P^WT^, *natR*), M-type strain (FS357; M^WT^, *kanR*) and M-type pheromone-less mutant (FS419; M^phe-less^, *hygR*). Cross-II, P-type strain (FS404; P^WT^, *natR*), M-type strain-1 (FS357; M^WT1^, *kanR*) and M-type strain-2 (FS423; M^WT2^, *hygR*). The same number of cells of the two M-type strains and twice the number of cells of the P-type strain were mixed. After incubation for 2 days, the cultures were diluted and then spread onto YEA plates containing 100 µg/ml of the appropriate drug: YEA+Nat, YEA+Nat+G418, and YEA+Nat+Hyg. After 3 days of incubation, the plates were photographed. The colony number on the plates was counted.

To confirm this result, we next performed a more quantitative assay. In this second experiment, three strains (wild-type P-strain, wild-type M-strain and M-factor-less M-strain) were differentially marked by three drug-resistance markers, *natR*, *kanR* and *hygR*, respectively. These three strains were mixed in SSL−N medium at the cell number ratio of 2:1:1. After incubation for 2 days, the cell suspension was spread onto YEA plates containing the appropriate drugs. Hybrid descendants between a wild-type strains conferred a *natR kanR* doubly resistant phenotype, whereas those between a wild-type P-strain and an M-factor-less M-strain carried the *natR hygR* trait. Colony numbers were counted and normalized to the number of *natR* colonies as a measure of wild-type P-cells. The frequency of the *natR kanR* recombinants appeared at about 5x10^−3^, whereas that of the *natR hygR* recombinants was <10^−6^. These results (Figure 7) indicate that wild-type P-cells preferentially mate with wild-type M-cells and only very rarely with M-factor-less M-cells. This tendency was not affected by the addition of exogenous M-factor (200 nM) to the SSL−N medium (Figure 7). In this experimental system, wild-type M-cells secreted M-factor pheromone in sufficient amounts for the completion of mating. Nevertheless, M-factor-less cells rarely participated in zygote formation. We conclude that only M-factor-producing M-cells are able to complete mating with P-cells.

A possible explanation for the above results is that mature M-factors or by-product peptides, made by proteolysis of the M-factor precursor protein, play an indispensable role in the mating capability of M-cells. The *mam1* ^*+*^ gene encodes an ABC transporter that is specific to M-factor secretion [35–37]. A *mam1Δ* mutant secretes no mature M-factor, and thus is sterile [35]. *mam1Δ* mutants may accumulate these hypothetical intracellular peptides. If these putative intracellular peptides were involved in zygote formation, then *mam1Δ* mutants (contrary to *mfm1,2,3Δ* mutants) would be able to mate with P-cells in the presence of exogenous M-factor. As shown in Supplementary Figure S1B, however, exogenous M-factor recovered significantly cell agglutination, but zygotes did not form. This observation indicates that secretion of M-factor is necessary for mating.

### No pheromone-induced polarized growth commences prior to cell-to-cell contact in liquid culture

**Figure 8:**
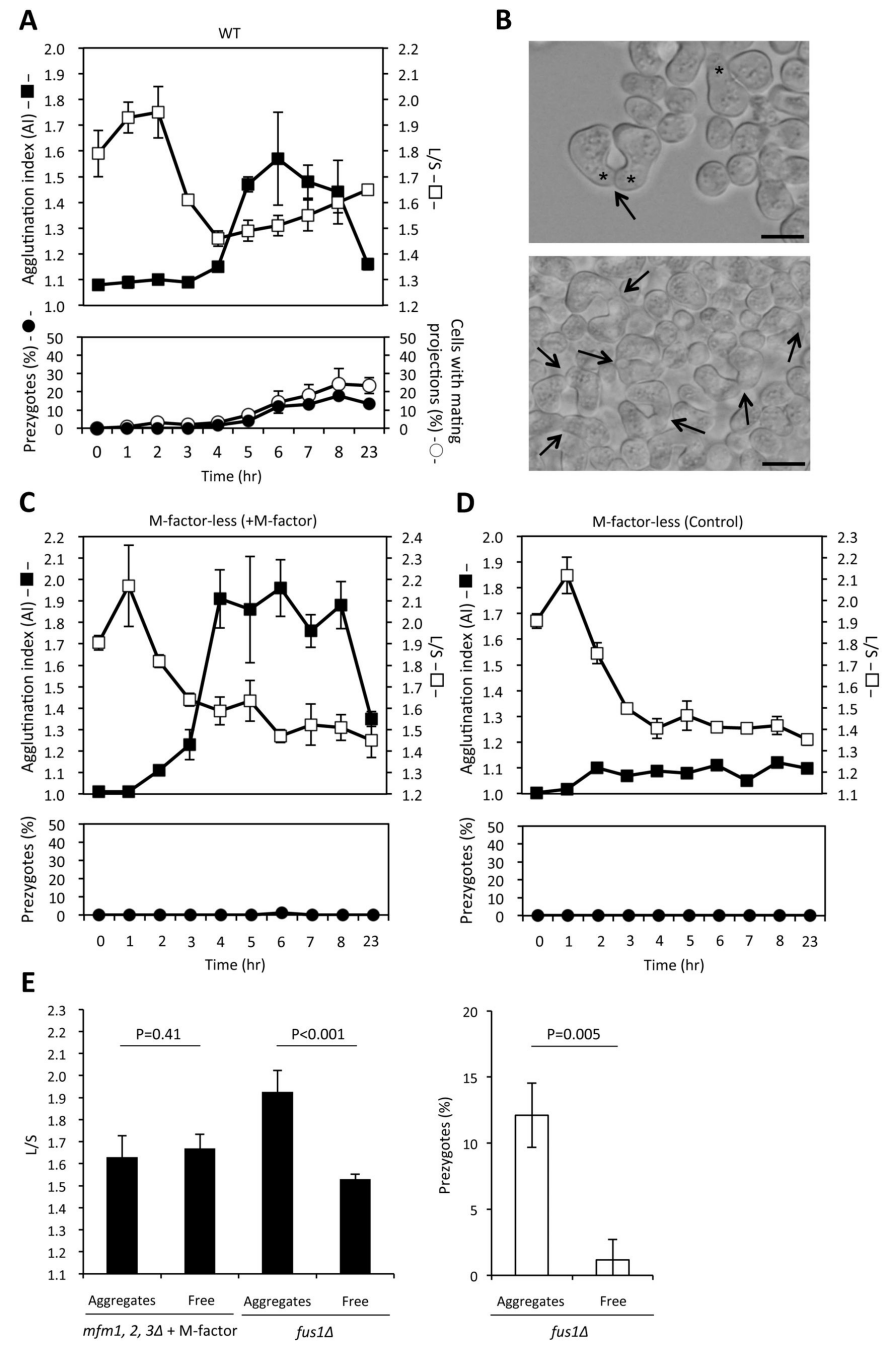
Kinetics of sexual agglutination and polar cell growth in liquid culture. (A) Mating kinetics of a homothallic wild-type strain (L968) cultured in SSL−N liquid medium. At hourly intervals, the intensity of agglutination (AI) was determined. Portions of the culture were subjected to brief sonication and microphotographs were taken. The long (L) and short (S) axes of cells were measured to calculate the L/S ratio for triplicate samples (100 cells each). The frequencies of prezygotes and cells with pointy projections were determined. (B) Microphotographs showing typical cell morphology. Arrows, prezygotes with a pointy mating projection (shown by asterisks). Scale bar, 10 µm. (C, D) Mating kinetics of M-factor-less mutant cells incubated with (C) or without (D) 200 nM M-factor. The experimental procedures are as described in (A). (E) Left, polar cell growth and prezygote formation in M-factor-induced aggregated cells and floating free cells. An M-factor-less strain (FS55) and an M-factor-producing *fus1Δ* strain (FS123) were incubated with 200 nM M-factor. After 4 hr, aggregates and free cells were isolated. After brief sonication, the L/S ratio was determined (n=1,000 for both strains). Right, frequency of prezygotes formed with *fus1Δ* (Aggregates *vs* Free: each *p*-value was shown, *t*-test)..

Cells placed on solid medium can sense a pheromone gradient and elongate a mating projection toward the strongest source of the pheromone [8]. The projection tip finally comes across the partner cell. After that, the cross walls dissolve to form a zygote. Under aqueous conditions, cell adhesion and polarized growth are induced by pheromone signaling. Because the exact timing of these two mating events has not been determined, we studied the kinetics of agglutination and polar cell growth in liquid culture. A wild-type homothallic strain (L968) was cultured in SSL−N liquid medium, and then the intensity of agglutination was determined and the morphology of cells was recorded. Polarized cell growth was evaluated by the ratio of the long axis (L) to the short axis (S) of each cell. Before cell fusion, a pair of mating partners firmly binds together at the projection tip. This pair of cells that is resistant to sonication is called the “prezygote” (see Figure 8B, arrows). Here, the frequencies of cells with a mating projection and those of prezygotes were determined. Cells underwent one or two round(s) of cell division without cell growth after inoculation into nitrogen-free liquid medium. As a result, the L/S ratio had remarkably decreased at 4 hr of incubation (Figure 8A). Cell agglutination took place almost simultaneously with an arrest in cell division after 4 hr of incubation. In turn, the L/S ratio began to increase after 5 hr of incubation, in accordance with the appearance of mating projections (Figure 8A). Mating projections and prezygotes were not observed before visible agglutination (AI of more than 1.1) (Figure 8A). We conclude that polarized cell growth does not take place before sexual agglutination commences. Finally, a pair of contacted cells, or a pair of sister cells in an aggregate, was traced by time-lapse microscopy until the cells came to fusion (Supplementary Figure S3). Several examples showed that cells extended mating projections at the contact site. These results support the idea that firm cell contact is attained before the start of cell elongation.

The kinetics of mating events was also examined with the M-factor-less mutant (FS55) cultured with or without M-factor. As shown in Figure 8D, neither agglutination nor prezygote formation occurred in the control culture without M-factor. When FS55 was treated with 200 nM synthetic M-factor, intense agglutination was detected after 3 hr of incubation (Figure 8C). Notably, the L/S ratio did not increase after 4 hr and no prezygotes were observed. These results indicate that for M-cells defective in M-factor production, the mating process is arrested before cell agglutination. When synthetic M-factor was supplied to the cells, agglutination was restored, but mating events remained arrested before polar cell elongation and prezygote formation.

Next, we compared polarized cell growth between aggregates and dispersed cells. The M-factor-less mutant (FS55) was incubated in SSL−N liquid medium containing 200 nM synthetic M-factor for 6 hr, when strong cell agglutination occurred. The culture was allowed to stand for a few minutes to allow the cell aggregates to sediment and then the aggregated cells and free dispersed cells were separately prepared. After sonication, cell size was measured to calculate the L/S ratio. As shown in Figure 8E, there was no difference in L/S ratio between the “aggregate fraction” and the “dispersed (free) cell fraction”. As a control, the same experiment was conducted with an M-factor-producing strain, FS123, harboring the *fus1Δ* allele. The *fus1Δ* mutant shows defects in cell fusion, but forms prezygotes [38]. The polarized growth was marked in the *fus1Δ* mutant, as compared with the wild-type strain. For the *fus1Δ* mutant, the L/S ratio was 1.3 times higher in aggregated cells than in free cells (Figure 8E, left). Furthermore, the frequency of prezygotes was also higher in the aggregates than in the free cells (Figure 8E, right). These results showed that polar cell elongation and prezygote formation were triggered after cell agglutination; as a result, cell-to-cell adhesion, commenced.

## Discussion

### A major target of the M-factor pheromone signal is the cell adhesion protein Map4

A wide variety of sex pheromones in animals serve to attract individuals of the opposite sex. Fungi including yeasts also release mating pheromones to enhance the chance of finding cells of the opposite sex [8]. Because yeasts are not motile, they are unable to move actively toward cells of the opposite mating type. Instead, yeast cells cultured on solid medium exhibit polarized growth toward cells of the opposite mating type that secrete mating pheromones [19,20,39]. It is interesting to understand the sexual behavior of yeasts in liquid medium. Fission yeast cells exhibit remarkable sexual agglutination in liquid mating culture [21]. Mating-type-specific cell adhesion results in the formation of marked cell aggregates, in which cells may find cells of the opposite mating type. The cell density of yeasts in nature is thought to be extremely low; thus, cell agglutination is important to enhance the chances of coming across cells of the opposite mating type. This might be the reason why mating pheromones primarily act to stimulate the sexual agglutinability between opposite mating-type cells.

Sexual cell agglutination in *S. pombe* requires both the P-type adhesin glycoprotein Map4 and its M-type counterpart, Mam3 [22,23]. We found that M-factor induces the cell adhesion activity of P-cells by inducing the production of Map4 (Figure 3). Although the downstream transcription factors of pheromone signals have not been identified, we noted two TR-box motifs in the *map4* promoter region (Figure 4). However, deletion of both TR-box motifs failed to completely inhibit the expression of *map4*, although *map4* expression was totally abolished in *ste11* deletion mutants (Supplementary Figure S2). Taken together, these facts suggest that *map4* ^*+*^ is indirectly controlled by Ste11. A few candidates for transcription factors downstream of Ste11 have been reported [40]. We found that M-factor treatment of *map4Δ* cells did not cause cell agglutination (Figure S1C). Conversely, ectopic overexpression of *map4* ^*+*^ caused agglutination without M-factor treatment (Figure 3C). These facts indicate that *map4* ^*+*^ expression is the key target of M-factor for agglutination.

### The M-factor pheromone has two different modes of action in the induction of mating ability and mate choice of P-cells

We found here that M-factor is essential for both sexual agglutination and the cell fusion process. Its counterpart P-factor was also essential for cell fusion and enhanced the agglutinability of M-cells. Although sexual agglutination was completely recovered by exogenous M-factor, the cell fusion defect was not suppressed. In addition, when M-factor-less M-cells were cultured with wild-type M-cells, the mutant cells were incorporated in the cell aggregates (Figure 5). Wild-type M-cells assisted the mutant M-cells by supplying M-factor pheromone, meaning that wild-type cells are altruistic and mutant cells behave as “cheats” in the sense of animal sociology. However, P-cells chose only wild-type M-cells as a mate to circumvent such cheating behavior. M-factor-less M-cells did not copulate with P-cells despite the presence of M-factor supplied by wild-type M-cells. Although the first step of mating—namely, cell-cell adhesion between different mating types—is controlled by diffusible M-factor peptide, uniform M-factor in the culture fluid was not sufficient to complete cell fusion. Pheromone-less cells are unable to transmit their own genes to the descendants, unless they incur the cost. An M-factor-specific ABC transporter, Mam1, is needed for cell fusion. Taken together, these observations indicate that the secretion of active M-factor is central to mate choice by P-cells.

In the present study focusing on mating in liquid culture, we proposed that cell-to-cell adhesion between different mating types triggered directional cell growth from the contact region. Pheromone-induced directed tip growth (termed “shmooing”) in the absence of the opposite mating-type cells has also been observed. P-type cells exhibit marked tip growth (shmooing) after treatment with high concentrations of exogenous M-factor (higher than 100 nM, data not shown) or after the accumulation of native M-factor in mating-deficient mutants such as *map4Δ* [23]. Such shmoo formation is caused by activation of the default polarization mechanism [19]. These results suggest that even in liquid culture, some cells can establish cell polarity without cell contact.

### Why M-factor-less cells are defective in cell fusion

A Rab family small G-protein, Cdc42, is a major polarity regulator in *S. pombe* [41]. Recently, Bendezu et al. [19] reported that the Cdc42/Scd1/Scd2 polarization complex migrated on the cell cortex in the presence of low concentrations of pheromone peptides (exploration stage), and the complex localized to a defined zone in response to a gradient of pheromones (commitment phase). Polarized cell growth is then commenced from the Cdc42 patch.

In a liquid culture with shaking, a pheromone gradient may not be stably formed, meaning that polarization must be established differently from the mechanisms proposed for mating on solid medium. Our findings suggested that a firm contact between a pair of M- and P-cells is crucial for polarized cell elongation (Figure 8). The concentration of pheromone peptides is probably higher in the cell contact region than in the external medium, because of the immediate perception of secreted pheromones. The retention of secreted pheromone peptides within the cell wall and periplasmic space may also play a role. We speculate that a Cdc42 polarization patch is locked at the close contact region from which the polarized growth initiates [19]. In fact, oriented cell extension took place after cells of opposite mating types came into close contact due to sexual cell adhesion. The thus-formed prezygotes become zygotes through complete lysis of the cross cell walls. On transfer of vegetative cells into nitrogen-free medium, cells undergo mostly two rounds of cell division without cell growth. During these residual cell divisions, mating-type interconversion takes place generating both P- and M-cells between which mating occurs [42]. A pair of daughter cells, one of which undergoes mating-type switching, fuse to form a zygote (sister-cell-mating). In this case, cells of a mating pair are separated only by a newly formed septum. Because M-factor-less mutants might not raise the concentration of pheromones in the contact region, the cells might not be able to establish the polarity necessary for mating. This model would explain why M-factor non-producing or non-secreting mutants are defective in cell fusion.

## Conclusion

**Figure 9:**
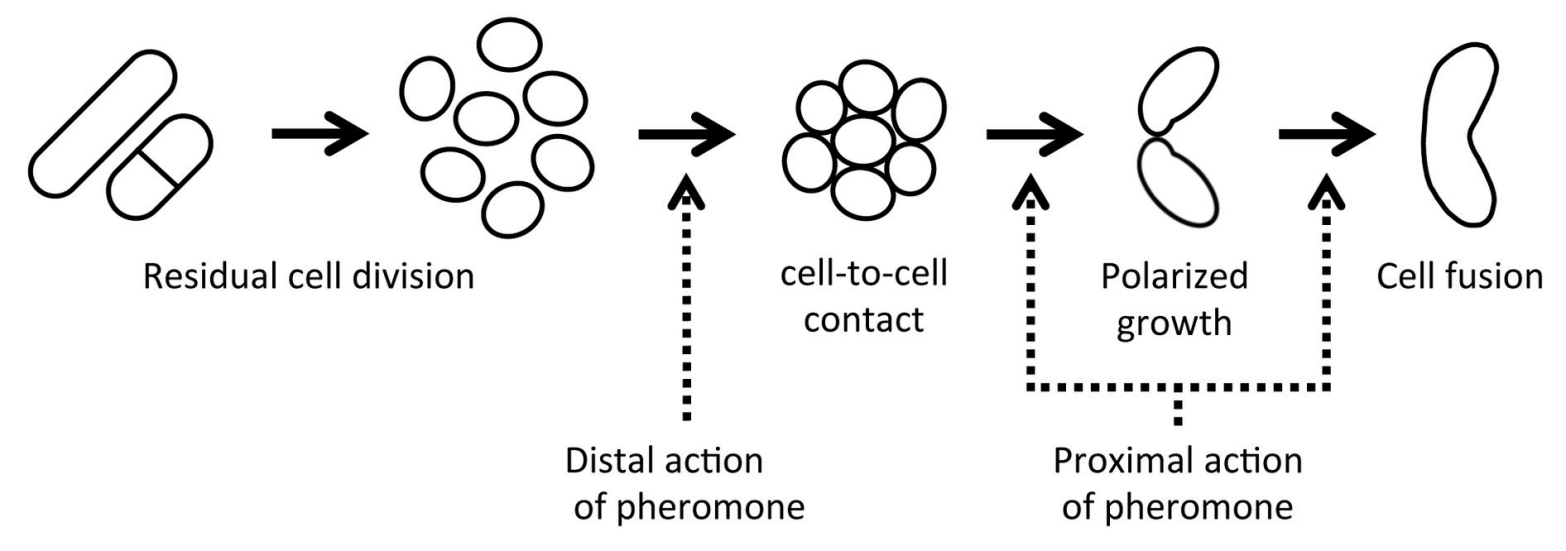
Illustration of the mating steps regulated by the distal and proximal actions of M-factor mating pheromone in liquid culture. When haploid cells of the opposite mating types are mixed in nitrogen-free liquid medium, cell agglutination occurs after arrest of cell division. Mating type-dependent cell-to-cell adhesion is induced in cell aggregates. From the contact site, polarized tip growth occurs leading to cell fusion. Pheromone is required for steps of cell agglutination (distal action), polarized growth and cell fusion (proximal action).

The mating pheromone M-factor has two different modes of action, namely a distal and proximal mode of action. Secreted, uniform M-factor induces sexual agglutinability of P-cells (distal action), which is essential for fixing the relative positions of M- and P-cells in liquid culture. Polarity establishment is attained by a more direct action of M-factor secreted by close neighbors (proximal action). Only M-cells that secrete M-factor can be chosen as a mate by P-cells. Mating steps in liquid culture regulated by these two different modes of pheromone actions are illustrated in Figure 9.

## Supporting Information

Figure S1 Effect of exogenous M-factor on cell agglutination and zygote frequency. The strains included a homothallic wild-type strain L968 (A), a homothallic *mam1Δ* strain Eg928 (B), and a homothallic *map4Δ* strain FS71 (C). Experimental procedures were followed as described in Figure 1B.
(TIF)

Figure S2 Expression of map4^PRO^-LacZ in *ste11Δ*. The plasmid pTA(map4^PRO^-lacZ) was introduced into an *h* ^+^ wild-type strain, FS85 (A) and the *h* ^+^ *ste11Δ* mutant, FS114 (B). The transformants were cultured in SSL+N lacking leucine, and then in SSL−N with or without 200 nM M-factor. Samples were taken every 2 hr and subjected to a β-galactosidase assay.
(TIF)

Figure S3 Time-lapse observation of the polarized tip growth of paired cells in aggregates. Cells of the homothallic wild-type strain (L968) were cultured in SSL−N for 4 hr. Aggregated cells were obtained and placed on an agarose slab gel containing SSL−N at 28^o^C. Observation was continued for the indicated duration (minutes) under an optical microscope. Frames were taken every 10 min. The arrow indicates a pair of cells, probably comprised of M- and P-type cells. Note that the cells were initially round in shape and protruded a pointy projection from the contact region. Scale bar, 10 µm.
(TIF)

Table S1 (DOCX)
