# Supplementary figures and images for: Distal and Proximal Actions of Peptide Pheromone M-Factor Control Different Conjugation Steps in Fission Yeast
Source: PLoS One. 2013 Jul 16;8(7):e69491. doi: 10.1371/journal.pone.0069491 (PMC3713066; doi:10.1371/journal.pone.0069491)

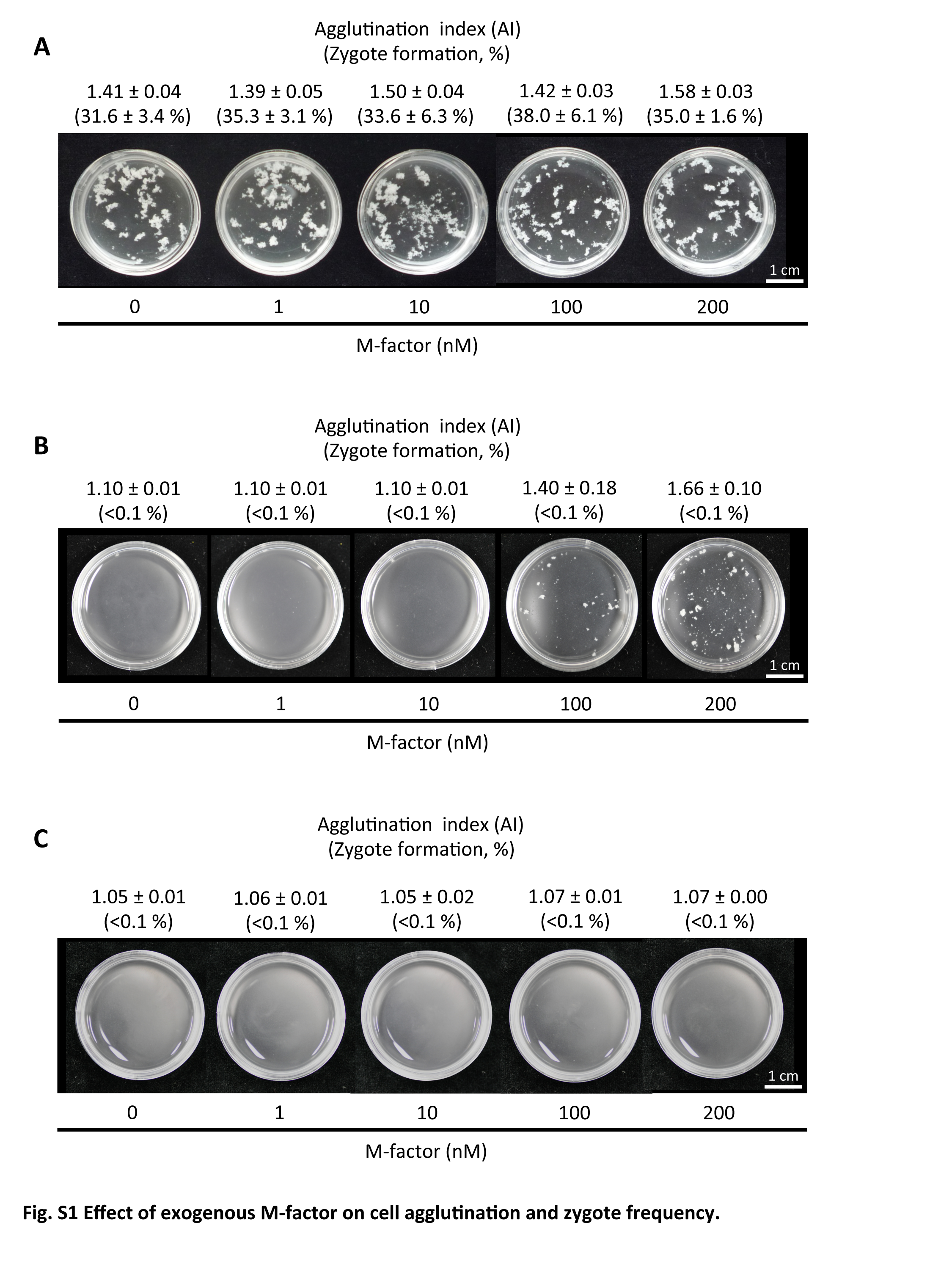

Supplement: Figure S1 — The strains included a homothallic wild-type strain L968 (A), a homothallic mam1Δ strain Eg928 (B), and a homothallic map4Δ strain FS71 (C). Experimental procedures were followed as described in Figure 1B. (TIF) [file pone.0069491.s001.tif]

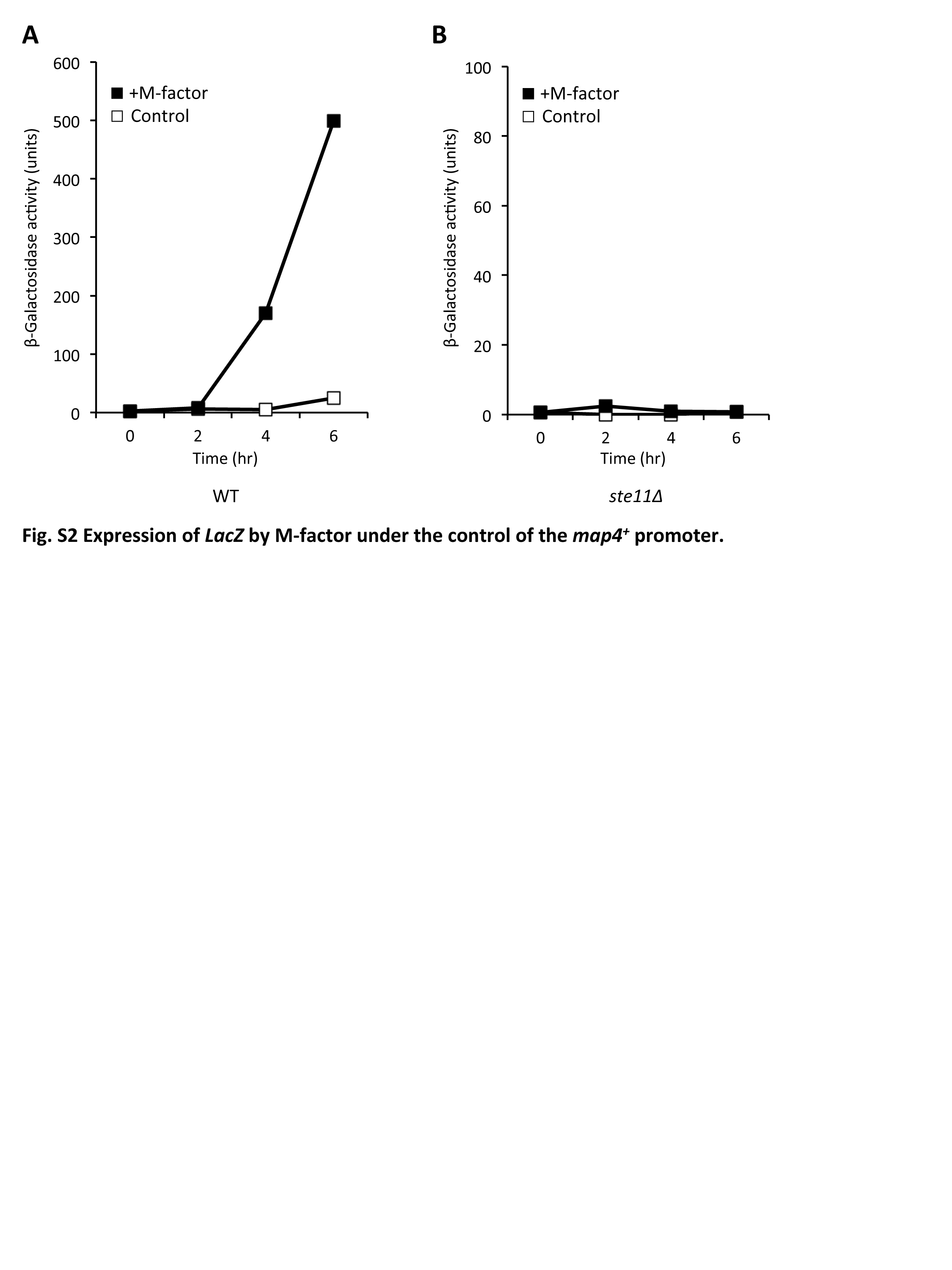

Supplement: Figure S2 — The plasmid pTA(map4PRO-lacZ) was introduced into an h + wild-type strain, FS85 (A) and the h + ste11Δ mutant, FS114 (B). The transformants were cultured in SSL+N lacking leucine, and then in SSL−N with or without 200 nM M-factor. Samples were taken every 2 hr and subjected to a β-galactosidase assay. (TIF) [file pone.0069491.s002.tif]

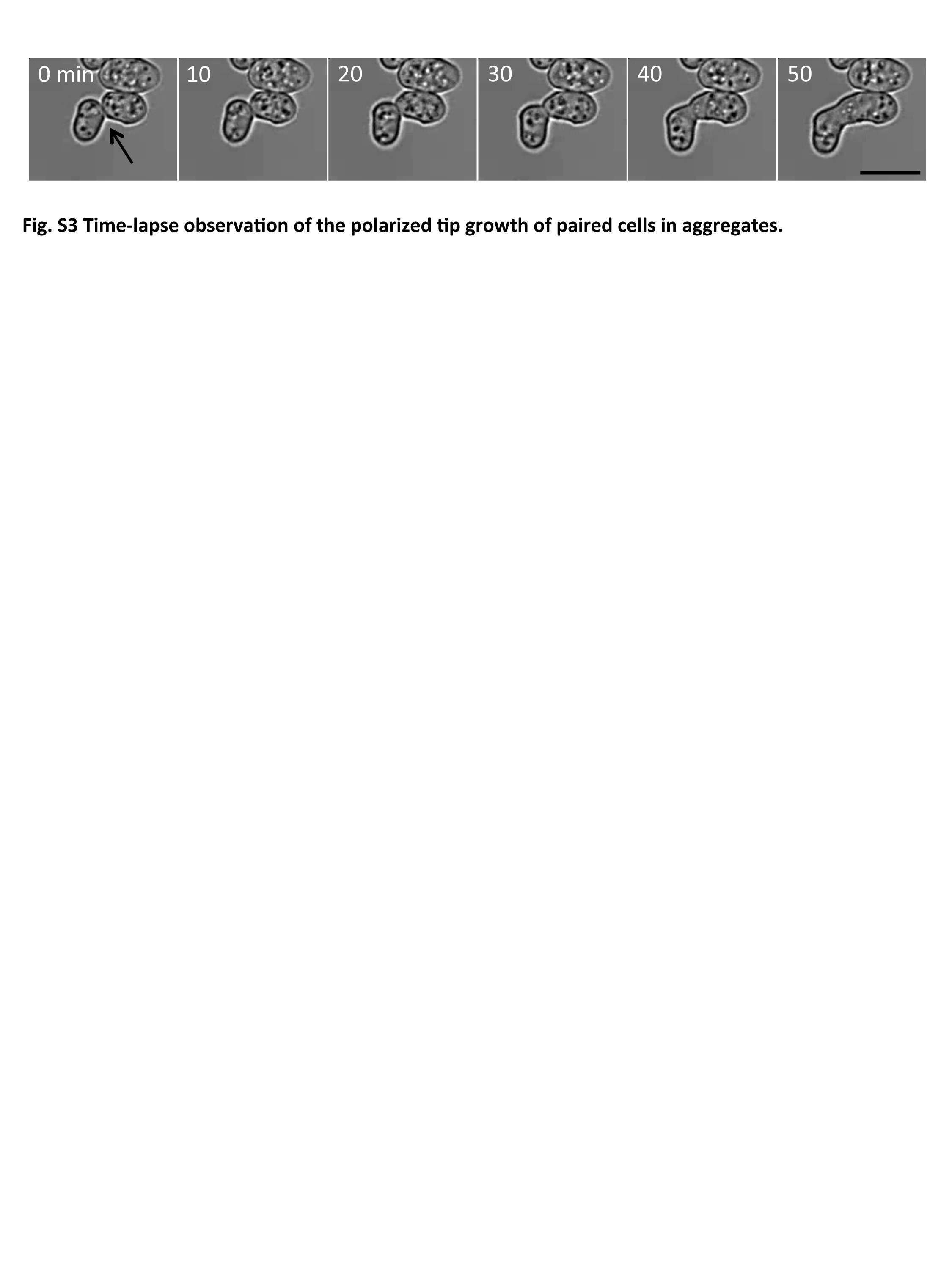

Supplement: Figure S3 — Cells of the homothallic wild-type strain (L968) were cultured in SSL−N for 4 hr. Aggregated cells were obtained and placed on an agarose slab gel containing SSL−N at 28oC. Observation was continued for the indicated duration (minutes) under an optical microscope. Frames were taken every 10 min. The arrow indicates a pair of cells, probably comprised of M- and P-type cells. Note that the cells were initially round in shape and protruded a pointy projection from the contact region. Scale bar, 10 µm. (TIF) [file pone.0069491.s003.tif]
