# Supplementary material for: Distal and Proximal Actions of Peptide Pheromone M-Factor Control Different Conjugation Steps in Fission Yeast
Source: PLoS One. 2013 Jul 16;8(7):e69491. doi: 10.1371/journal.pone.0069491 (PMC3713066; doi:10.1371/journal.pone.0069491)
Supplement: Table S1 — (DOCX) [file pone.0069491.s004.docx]

Supplementary Table

Table S1. Primers used for amplification of the *map4* promoter

| **Primers** | **Amplified segment** | **Nucleotide sequence*** |
| --- | --- | --- |
| F1H | Full length of the promoter | ggaagcttaaatgagcttatgtgtttag (*Hind*III) |
| R1X | Full length of the promoter | ccctcgagcaacaaagaacattgagtcg (*Xho*I) |
| R2X | For 3’ small deletion | ccctcgagtcgaaagaatttcacaaatgc (*Xho*I) |
| R3X | For 3’ large deletion | ccctcgagataagcatccatttttggtagc (*Xho*I) |
| F2H | For 5’ large deletion | ggaagcttaagtttagacgtgtgtccacc (*Hind*III) |

*Appended restriction sites are underlined. Restriction enzymes are shown in the parentheses.
